# Meeting of the American Society Dental Surgeons, West Point, August 2, 1853

**Published:** 1853-10

**Authors:** 


### ARTICLE XII.

### *Meeting of the American Society Dental Surgeons, West Point, August* 2, 1853.

THE American Society of Dental Surgeons convened at West Point at 12 o'clock. President Dr. E. Parmly in the Chair, Dr. C. O. Cone, Secretary.

The minutes of the last annual meeting were read by the secretary, and approved.

The chairman of the executive committee made a report to govern the doings of this meeting of the association for this session.

The treasurer presented his report for the past year.

The society then adjourned to meet at 9 A. M. on the 3d August.

*Wednesday, August* 3, 9 A. M. The society was called to order agreeable to adjournment, by the president.

The treasurer's report was read, and referred to a committee. Dr. E. Townsend, Jos. H. Foster.

The committee on microscopic observations of the characteristics of saliva, and the secretions of the mouth, reported progress, and was continued. Drs. E. Townsend, J. D. White.

The committee on foreign dental literature was continued in the absence of the chairman, Dr. C. A. Harris.

On motion of Dr. E. Townsend,

*Resolved*, That invitation be extended to all members of the profession present at West Point, to join us in our sessions—a committee of one was appointed to carry it into effect. Dr. Jos. H. Foster.

On motion of Dr. Jos. H. Foster,

*Resolved*, That in consequence of the absence of Dr. S. P. Hullihen, the order of business be so far changed, that the essay of Dr. E. Townsend take the place of the opening address.

Dr. E. Townsend then addressed the society on the unprofessionality of dental patents, at the conclusion of which, the committee to revise the constitution and by-laws, reported through their chairman, Dr. J. D. White, a code of laws for their future government.

On motion of Dr. E. Townsend, the report was accepted, and the committee discharged from further duty.

On motion of Dr. Bonsall, the constitution was adopted, article by article; and after some discussion, the constitution as amended, was adopted.

The by-laws were then acted upon in a similar manner and adopted. The society then adjourned to meet at 4 o'clock, P. M.

*Afternoon Session.* Patrick Houston's resignation was then presented and accepted.

Dr. E. J. Dunning then drew the attention of the society to a fulsome and offensive advertisement of one of its members.

On motion of Dr. J. D. White,

*Resolved,* That any member of this society who shall extol his own superior merits over a fellow practitioner in the public prints, or employs means of advertisement which may be regarded by the society as lowering the dignity of the profession, or compromising its character, shall be impeached, suspended, or expelled. Adopted.

*August 4th, Morning Session.*—Society met agreeably to adjournment, and was called to order by the president, Dr. E. Parmly.

On motion of Dr. Chas. Bonsall, seconded by E. G. Tucker,

*Resolved,* That inasmuch as a pamphlet advertisement of Dr. Thomas Palmer, of Fitchburg, Mass., a member of this society, has been laid before us, which is considered highly reprehensible and derogatory to the character of the association and the profession; therefore,

*Resolved,* That the said Dr. Thos. Palmer be, and is hereby suspended from membership.

On the motion of Dr. Bonsall, a discussion ensued by Drs. Tucker, Dunning, Miller, J. Parmly and Townsend.

On motion of Dr. Townsend, seconded by Dr. Dunning,

*Resolved*, That Dr. Palmer be notified of the action of the society, and a copy of the law by which he was suspended transmitted to him.

On motion of Dr. Cone, seconded by Dr. Dunning, Dr. Foster was appointed secretary *pro tem.*

The committee appointed upon motion of Dr. Goddard made the following report, which was adopted.

On motion of Dr. E. Townsend, seconded by Dr. J. D. White,

*Resolved*, That the society proceed first to the election of the executive council and examining committee—adopted—and resulted as follows: Drs. E. Townsend, J. D. White, C. C. Williams, Joshua Tucker, A. C. Hawes.

On motion of Dr. E. Townsend, seconded by Dr. C. O. Cone,

*Resolved*, That the election of officers proceed in the manner in which they are placed in the order of business.

Dr. E. Parmly being nominated for president, declined, for urgent reasons, serving the society longer in that capacity.

Drs. E. Townsend, J. H. Foster and J. D. White were then nominated—the two latter declined serving—and on the first ballot Dr. E. Townsend was unanimously elected.

Dr. J. H. Foster, Dr. Joshua Tucker and Dr. W. H. Goddard were unanimously elected vice-presidents.

Election of corresponding and recording secretary being next in order, Dr. Cone was nominated, but peremptorily declined serving, Dr. Dunning also declined. Dr. David R. Parmly was then nominated and declined, but the society persevered in the ballot, when upon an unanimous election, he consented to serve the association for one year.

Dr. E. J. Dunning, was then elected treasurer. Dr. D. R. Parmly elected librarian.

Committee of publication, Drs. J. D. White, E. Townsend, D. R. Parmly.

Committee to take cognizance of improvements in the profession, and report at next meeting, Drs. E. G. Tucker, Jahial Parmly, J. D. White.

Dr. Jahial Parmly was appointed to deliver the opening address at the next annual meeting.

The following members were then appointed to prepare essays on some subject connected with the theory or practice of dental surgery, to be read before the next annual meeting, Drs. A. Robertson, A. C. Hawes, A. Westcott, C. C. Williams, E. G. Tucker.

On motion of Dr. W. H. Goddard, seconded by Dr. C. Bonsall,

*Resolved,* That when this society adjourn, it shall hold its next annual meeting at Cincinnati, Ohio, on the first Tuesday in August, 1854, at such hour and place as shall be designated by the secretary.

On motion of Dr. Dunning, seconded by Dr. Bonsall,

*Resolved,* That the thanks of the society be expressed to Dr. E. Townsend, for his able address read before the society yesterday, and that he be requested to furnish it to the secretary for publication. Adopted.

*Afternoon Session.*—The society was called to order. Dr. E. Townsend, in the chair.

The following gentlemen having been examined by the committee, and reported as worthy of membership, was unanimously elected by the society: Dr. R. P. Berry, of Newport, R. I.; Dr. J. S. Clark, D. D. S., of New Orleans, La.; Dr. Francis Field, Waltham, Mass.; Dwight Tracy, M. D., D. D. S., Willimantic, Conn.

The society then adjourned to meet first Tuesday in August, 1854, at Cincinnati, Ohio.

